# Four-dimensional noise reduction using the time series of medical computed tomography datasets with short interval times: a static-phantom study

**DOI:** 10.7717/peerj.1680

**Published:** 2016-02-09

**Authors:** Tatsuya Nishii, Atsushi K. Kono, Wakiko Tani, Erina Suehiro, Noriyuki Negi, Satoru Takahashi, Kazuro Sugimura

**Affiliations:** 1 Department of Radiology, Kobe University Graduate School of Medicine, Kobe, Japan; 2 Division of Radiology, Center for Radiology and Radiation Oncology, Kobe University Hospital, Kobe, Japan

**Keywords:** Image quality, Computed tomography, Radiation dose, Temporal noise reduction, Cardiac CT

## Abstract

**Backgrounds.** This study examines the hypothesis that four-dimensional noise reduction (4DNR) with short interval times reduces noise in cardiac computed tomography (CCT) using “padding” phases. Furthermore, the capability of reducing the reduction dose in CCT using this post-processing technique was assessed.

**Methods.** Using base and quarter radiation doses for CCT (456 and 114 mAs/rot with 120 kVp), a static phantom was scanned ten times with retrospective electrocardiogram gating, and 4DNR with short interval times (50 ms) was performed using a post-processing technique. Differences in the computed tomography (CT) attenuation, contrast-to-noise ratio (CNR) and spatial resolution with modulation transfer function in each dose image obtained with and without 4DNR were assessed by conducting a Tukey–Kramer’s test and non-inferiority test.

**Results.** For the base dose, by using 4DNR, the CNR was improved from 1.18 ± 0.15 to 2.08 ± 0.20 (*P* = 0.001), while the CT attenuation and spatial resolution of the image of 4DNR did not were significantly inferior to those of reference image (*P* < 0.001). CNRs of the quarter-dose image in 4DNR also improved to 1.28 ± 0.11, and were not inferior to those of the non-4DNR images of the base dose (*P* < 0.001).

**Conclusions.** 4DNR with short interval times significantly reduced noise. Furthermore, applying this method to CCT would have the potential of reducing the radiation dose by 75%, while maintaining a similar image noise level.

## Introduction

Four-dimensional noise reduction (4DNR) is a powerful post-processing method of reducing noise by employing spatial–temporal analysis. Large noise reduction using time analysis is well known in other fields such as video processing. Noise and artifacts can appear randomly in the temporal direction. By adopting temporal filtering with an appropriate estimation of motion (Brailean et al., 1995), the effect of random noise is minimized.

Several filtering methods have been recently proposed for the 4DNR of medical images (Liu et al., 2009) and can be used for a four dimensional dataset with any medical imaging modality such as CT. For the CT datasets, because not only the volume dataset but also temporal axis data are required, only multi-data acquisition examination has been considered for the application of 4DNR. Thus, 4DNR is generally performed for time series of volume datasets having a relatively long interval time (e.g., 1000 ms), such as datasets of computed tomography (CT) perfusion examinations (Li et al., 2014a; Li et al., 2014b; Corcuera-Solano et al., 2014). However, the applicability of 4DNR to time series of CT volume datasets with short interval times has not been fully discussed (Otton et al., 2013; Tatsugami et al., 2015). In contrast to the case for datasets having long time intervals, the strong advantage of applying 4DNR in the case of short interval times is considered to be the ability to scan datasets constructed by single data acquisition. Thus, we developed a method of applying 4DNR to datasets having short interval times (≤50 ms), which we refer to as the method of obtaining a noisele ss imag e by a daptive phase-shifted to pological coherence analysis or *legato*.

Cardiac CT (CCT) usually acquires datasets that include “padding” phases centered on the mid-diastole. From these “padding” phases, time-series datasets with short interval times can be easily reconstructed (Tatsugami et al., 2015). However, the additional information provided by additional phases has largely been ignored in post-processing noise reduction for CCT. *Legato* can be applied to such datasets to reduce noise and the radiation dose in CCT. The present study conducts quantitative image quality analysis using static *ex vivo* phantoms and *in vivo* retrospective analysis to examine the hypothesis that post processing with *legato* reduces the noise in CCT images and permits a lower radiation dose when using “padding” phases.

## Materials and Methods

### Study design

The present study comprises from three *ex vivo* and four *in vivo* studies. The following four analyses was performed; (1) the *ex vivo* preliminary analysis of *legato*, (2) the *ex vivo* quantitative analysis of images post processed with *legato*, (3) the *ex vivo* assessment of the ability to reduce the radiation dose using *legato*, and (4) the *in vivo* assessment of the ability to reduce the radiation dose using *legato.*

### Image acquisition and reconstruction

Images were acquired using a 128-detector row dual-source CT device (SOMATOM Definition Flash, Siemens AG, Forchheim, Germany).

#### Ex vivo study

Complete calibration scanning was performed before phantom data were acquired. Retrospective electrocardiogram (ECG)-gated helical scans were made of a Catphan phantom (The Phantom Factory, Salem, NY, USA), wire phantom (Quality Phantom Set, Siemens AG, Forchheim, Germany), and 330-mm-diameter water phantom in electrocardiography simulation mode (60 bpm). Each phantom was scanned ten times. The detailed parameters are given in Table 1. In the case of scanning the Catphan phantom, two tube current-time products were used; i.e., 456 mAs/rot as a reference radiation dose and 114 mAs/rot as a quarter radiation dose. In the case of scanning the wire and water phantom, a current-time product of 456 mAs/rot was used. The Catphan and water phantoms were positioned at the isocenter of the gantry. The wire phantom was slightly offset from the isocenter of the gantry (Ichikawa et al., 2008).

**Table 1:** Data acquisition parameters.

	*Ex vivo*			*In vivo*
	Catphan	Wire	Water	
**Image acquisition**				
Collimation (mm)		0.6		
Rotation speed (ms/rot)		280		
Tube voltage (kVp)		120		
Helical pitch		1.7		
Tube-current time products (mAs/rot)	456, 114	456	456	AEC:ref. 390 mAs (CARE Dose4D)
**Image reconstruction**		Half		
Slice thickness (mm)		1		3
Slice interval (mm)		1		3
Field of view (mm)	250	50	200	240
Kernels	FBP(B26f), IR(I26f)	FBP(B26f)	FBP(B26f)	IR(I26f)
Cardiac phase (ms from R wave)	−300, −250, −200	−300, −250, −200	−300, −250, −200	−800, −750, −700, −250

**Notes.**

AEC auto exposure control ref. reference mAs FBP filtered back projection IR iterative reconstruction

For the *ex vivo* preliminary analysis, three phases separated by intervals of 10, 30, 50, 70 and 90 ms (each center phase was set as −250 ms relative to the R wave) were reconstructed with convolution kernels for assessment of the coronary artery employing filtered back projection (FBP) (B26f).

For the other two *ex vivo* studies, three phases separated by intervals of 50 ms (−300, −250, −200 ms relative to the R-wave) were reconstructed with convolution kernel employing FBP and iterative reconstruction (IR) (SAFIRE, Sinogram Affirmed Iterative Reconstruction, Siemens AG, Forchheim, Germany) with strength 3 (B26f and I26f, respectively). The interval times were decided by the result of the *ex vivo* preliminary study, that the CNR reached a plateau at an interval time of 50 ms.

#### In vivo study

Fifteen consecutive cases (mean age of 66.3 years; age range of 45–84 years; two females and 13 males) who underwent retrospective ECG-gated helical CCT examination with dose modulation mode in October to November 2014 were enrolled in the present study. Our institutional review board approved the study (No. 1372). Written informed consent from all subjects was waived by our institutional review board because of the retrospective nature of the study. Employing the dose modulation mode, a data acquisition was conducted using a full dose in the diastolic phase while a quarter dose was used for the systolic phase. The parameters for image acquisition are given in Table 1. If the heat rate was higher than 75 bpm, a beta-blocker was used prior to the examination. Iopamidol (Iopamiron 370; Bayer Yakuhin, Osaka, Japan) was injected at a concentration of 370 mgI/mL via a 22-gauge catheter into the right antecubital vein at a flow rate of 22 mgI/s/kg over a period of 15 s, which was followed by a saline flush of 30 mL at the same rate. Bolus tracking was performed for a region of interest (ROI) in the ascending aorta. The scan automatically started 6 s after contrast enhancement of the ROI reached a threshold of +150 Hounsfield units (HU). The image dataset for the mid-diastolic phase (−250 ms relative to the R-wave) obtained with the reference dose and three image datasets for the systolic phase (200, 250, 300 ms relative to the R-wave) obtained with the quarter dose were reconstructed with the parameters given in Table 1.

### Image post processing

Two board-certified Roentgen technologists who were blinded to the subjects’ identities performed further post image processing and image analyses. For the post processing including the implementation of *legato*, a commercially available workstation (Ziostation 2.1.7.1 and PhyZiodynamics Technology, Ziosoft Inc., Tokyo, Japan) was used (Wai et al., 2013; Koike et al., 2014). From the three collected timeline datasets, three computed timeline datasets were calculated using ×1 for the generation of interpolated phases, a weight of 0.3 with two phases, and a non-cyclic algorithm as parameters, which we can set for the 4DNR with PhyZiodynamics Technology. PhyZiodynamics is software for non-rigid registration-based noise reduction, motion coherence and functional analysis. Noise is reduced through voxel-to-voxel mapping by tracking the temporal and spatial movement of individual voxels according to registration and interpolation algorithms (Brown, 2010). Image datasets for the center phase obtained from the collected and computed datasets are referred to as non-*legato* and *legato* images, respectively, and used in the following image analyses.

### Image analysis

#### Ex vivo preliminary analysis of legato

The non-*legato* images scanned at 456 mAs/rot were set as reference images. The contrast-to-noise ratio (CNR) of each *legato* image with several interval times was obtained. The CNR assessments were performed using Module CTP515 of Catphan and Image J (Schneider, Rasband & Eliceiri, 2012). Circular ROIs were set for the 0.1%, 10-mm module and the neighboring background to obtain the mean and standard deviation (SD) of the CT attenuation within the ROI. The CNR was calculated as CNR = (ROI_T_ − ROI_B_)/SD_B_, where ROI_T_ is the mean attenuation for the target module, ROI_B_ is the mean attenuation for the background, and SD_B_ is the SD of the background. In addition, to assess the difference between two-phase images for each interval form 10 to 90 ms, the mean squared error (MSE) (Zhou & Bovik, 2009) of each pixel was calculated using Image J.

#### Ex vivo quantitative image analysis of legato

The non-*legato* images scanned at 456 mAs/rot were set as reference images. The *legato* images obtained with the same radiation dose were compared with the reference image in terms of the CT attenuation, CNR, modulation transfer function (MTF), and noise power spectrum (NPS).

The CT attenuation and CNR were assessed for the images of Module CTP515 in Catphan. Both FBP and IR images were assessed. The measurement and calculation of the CT value and CNR were the same as previously described.

To assess the spatial resolution of an image, MTF and MTF_10%_ were calculated employing the wire method and the software CTmeasure (http://www.jsct-tech.org/, 2012–2014) from the FBP image of the wire phantom.

To examine the variance and spatial frequency characteristics of image noise, the NPS was measured from the FBP image of the water phantom employing CTmeasure and the radial frequency.

#### Ex vivo assessment of the ability to reduce the radiation dose using legato

The CT value and CNR were assessed for the images of Module CTP515 in Catphan acquired at the reference radiation dose, quarter radiation dose, and quarter radiation dose using *legato*. The image datasets obtained with IR were used. The measurement and calculation of the CT number and CNR were the same as previously described. Furthermore, two board-certified radiologists (TN, with 7 years’ experience and AKK, with 12-years’ experience) was assessed the subjective image quality analysis by consensus reading in a blind manner. The image score was defined as the number of the modules that we could detect in each image with a slice thickness of 5 mm.

#### In vivo assessment of the ability to reduce the radiation dose using legato

Images of the diastolic phase (reference radiation dose) obtained without *legato* and the systolic phase (quarter radiation dose) obtained with and without *legato* were assessed. The circular ROI was set as the descending aorta at the level of the left atrium. The noise level as the SD of CT attenuation within the ROI was recorded.

### Statistical analysis

The mean and SD values of metric variables were analyzed. Differences were assessed conducting a Tukey–Kramer’s honestly significant difference test. For the equivalence test, both bounds of 95% confidence intervals (CIs) of the difference from the reference image were compared with a margin set of a CT value of 2 HU. In the non-inferiority test, the lower bounds of 95% CIs of the difference from the reference image were compared with a margin set of a CNR of 0.1, MTF_10%_ of 0.05 cycles/mm, and SD of 1 HU. The significance level was set at *P* = 0.05.

For statistical analysis, the software JMP9.0 (SAS Institute Japan, Tokyo, Japan) and R (R Foundation for Statistical Computing, Vienna, Austria) was used.

## Results

### *Ex vivo* preliminary analysis of *legato*

**Figure 1:**
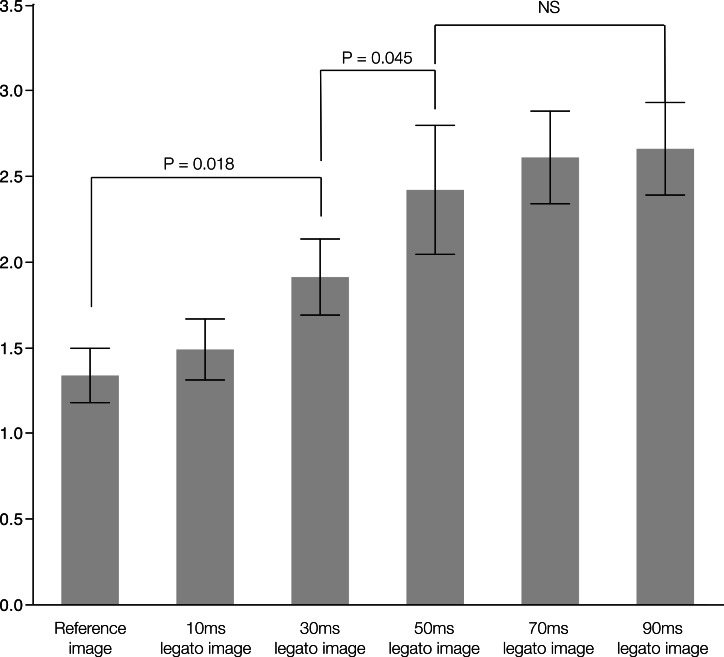
The relationship with contrast noise ratio and interval times for *legato*. *Legato*, the method of obtaining a noisele ss imag e by a daptive phase-shifted to pological coherence analysis.

The CNR improved with an increase in interval time from 10 to 50 ms. However, the CNR reached a plateau at an interval time of 50 ms, beyond which it did not change considerably (Fig. 1). The CNRs were 1.33 ± 0.16 for the reference image, 1.48 ± 0.18 for an interval time of 10 ms, 1.90 ± 0.22 for 30 ms, 2.41 ± 0.38 for 50 ms, 2.61 ± 0.27 for 70 ms, and 2.65 ± 0.27 for 90 ms. In addition, the MSE for each interval time of 10, 30, 50, 70, and 90 was 14.0 ± 0.7, 64.9 ± 4.5, 114.2 ± 5.1, 151.0 ± 5.8, and 149.3 ± 4.9 respectively. Although, the MSE increased significantly with the interval time until 70 ms (*P* < 0.001), the MSEs for intervals of 70 and 90 ms were not significantly different.

### *Ex vivo* quantitative image analysis of *legato*

**Figure 2:**
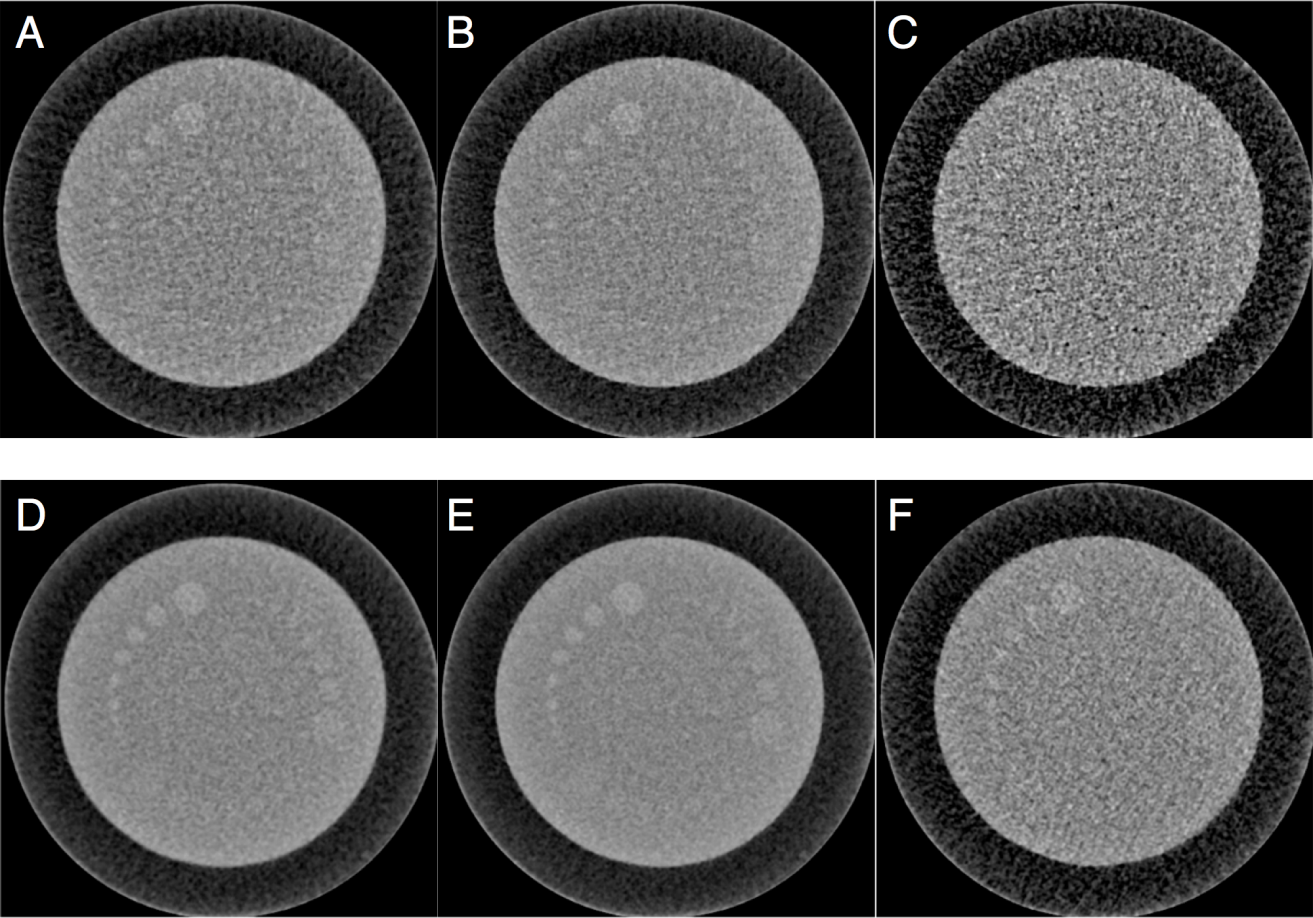
Reference and *legato* images. The original images obtained by filtered back projection (FBP) with reference dose (A), iterative reconstruction (IR) with reference dose (B), and IR with quarter radiation dose (C) are shown in upper column. By using *legato*, the image noise levels are significantly improved in images of FBP with reference dose (D), IR with reference dose (E), and IR with quarter radiation dose (F). *Legato*, the method of obtaining a noisele ss imag e by a daptive phase-shifted to pological coherence analysis.

**Figure 3:**
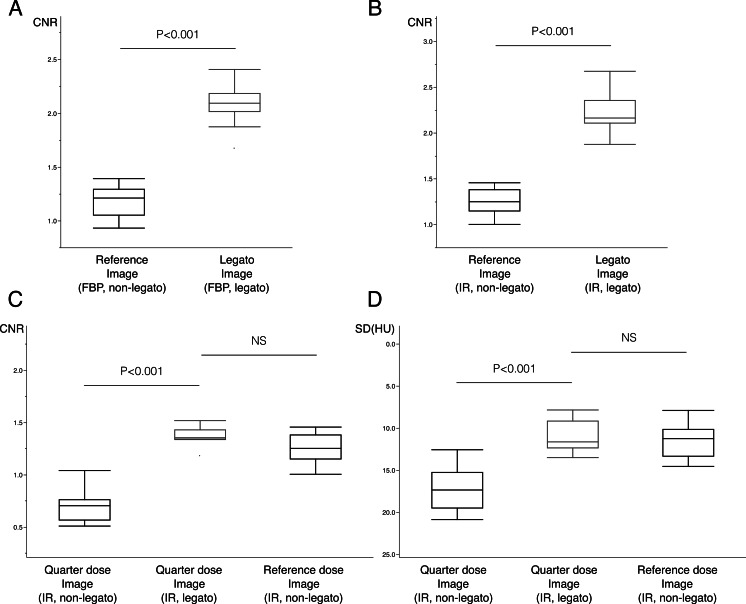
Box plots of the contrast-noise ratio (CNR) and image noise. Significant improvements in the CNRs of the images obtained by filtered back projection (FBP) (A), iterative reconstruction (IR) (B), quarter dose image-acquisition (C), were achieved using *legato*. Significant noise reduction of the *in-vivo* image with quarter dose (D), were also achieved using *legato*. *Legato*, the method of obtaining a noisele ss imag e by a daptive phase-shifted to pological coherence analysis.

The CNR obtained with *legato* was significantly better than that of the reference image (1.18 ± 0.15 versus 2.08 ± 0.19, *P* < 0.001) (Figs. 2A, 2B and 3A). Even when employing the IR method, significant noise reduction was possible with *legato* (1.25 ± 0.15 versus 2.21 ± 0.22, *P* < 0.001) (Figs. 2C, 2D and 3B). In contrast, the CT attenuation did not change significantly when using *legato* (Table 2).

**Table 2:** Results on the equivalence test and non-inferiority test.

	Reference	*Legato*	Margin	Difference [95% CI]	*P* value
CT value (HU)					
FBP	63.4 ± 0.6	63.4 ± 0.4	2	−0.02 [−0.51–0.46]	<0.001
IR	63.6 ± 0.6	63.9 ± 0.4	2	−0.05 [−0.57–0.47]	<0.001
MTF_10%_ (cycles/mm)	0.55 ± 0.01	0.56 ± 0.02	0.05	0.005 [−0.010–0.019]	<0.001

	Reference	Quarter dose +*legato*	Margin	Difference [95% CI]	*P* value
***Ex vivo***					
CNR	1.25 ± 0.15	1.37 ± 0.09	0.1	0.113 [−0.003–0.230]	<0.001
CT value (HU)	63.6 ± 0.6	63.9 ± 0.9	2	−0.3 [−0.96–0.40]	<0.001
***In vivo***					
SD (HU)	11.46 ± 1.99	11.01 ± 1.84	1	−0.455 [−1.89–0.980]	0.027

**Notes.**

FBP filtered back projection IR iterative reconstruction CI confidence interval MTF modulation transfer function SD standard deviation *Legato* the method of obtaining a noisele ss imag e by a daptive phase-shifted to pological coherence analysis

**Figure 4:**
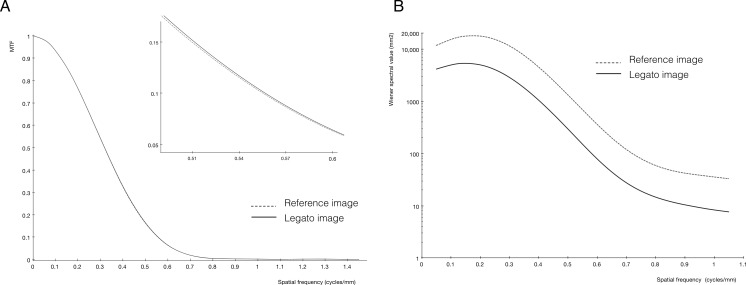
Results of modulation transfer function (MTF) analyses and noise power spectrum (NPS) analysis. The MTF curve (A) was almost the same for the image processed with *legato* (black line) and the reference image not processed with *legato* (gray dot line). The NPS curve (B) of the *legato* image (black line) shows the uniform reduction in noise throughout the frequency band relative to the noise level in the reference image (gray dot line). *Legato*, the method of obtaining a noisele ss imag e by a daptive phase-shifted to pological coherence analysis.

In the assessment of spatial resolution, MTF_10%_ obtained with *legato* was not inferior to that of the reference image (Table 2). Each MTF curve is shown in Fig. 4A.

According to NPS analysis shown in Fig. 4B, noise was reduced equally throughout the frequency band.

### *Ex vivo* assessment of the ability to reduce the radiation dose using *legato*

With *legato*, CNRs for the quarter radiation dose significantly improved from 0.71 ± 0.15 to 1.37 ± 0.09 (*P* < 0.001) (Figs. 2E and 2F) and were not inferior to those of the reference dose image (Table 2 and Fig. 3D). CT attenuation did not differ among reference dose image, quarter radiation dose image, and quarter radiation dose image with *legato* (63.6 ± 0.6 HU, 63.7 ± 0.5 HU and 63.9 ± 0.9 HU, respectively, *P* < 0.001 for each). Furthermore, the subjective image score had a median value of 16 (range 13–18) for the reference, 6 (range 4–9) for the quarter radiation dose, and 15 (range 13–18) for the quarter radiation dose with *legato*.

#### *In vivo* assessment of the ability to reduce the radiation dose using *legato*

**Figure 5:**
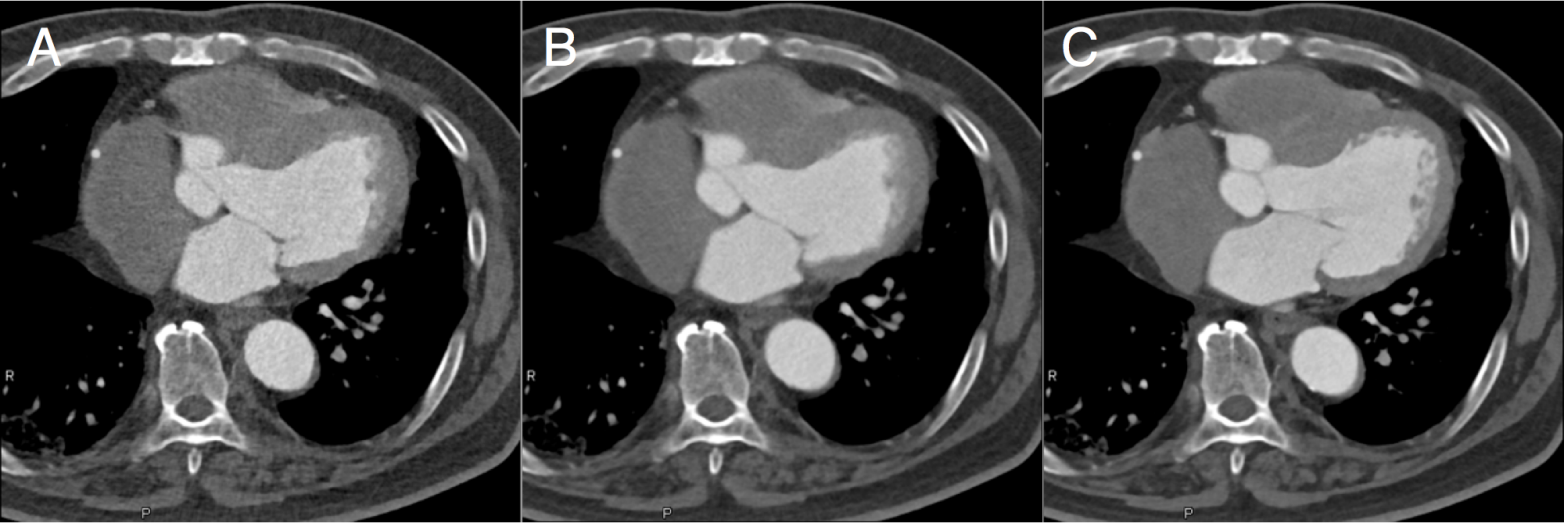
Representative images of *in-vivo* study. Representative clinical images are shown; from the left, a quarter-radiation-dose image (systolic phase) without *legato* post processing (A), a quarter-radiation-dose image (systolic phase) with *legato* post processing (B), and a reference-dose image without *legato* post processing (C). *Legato*, the method of obtaining a noisele ss imag e by a daptive phase-shifted to pological coherence analysis.

The details of the subjects in the *in vivo* study are listed in Table 3. The *legato* images were obtained successfully and the post-processing time was approximately 10 min in each case. A representative image for the case of a 70-year-old male is shown in Fig. 5. The noise level in the image acquired at a quarter radiation dose and processed with *legato* was significantly lower than that in the image not processed *legato* (17.11 ± 2.73 versus 11.01 ± 1.84, *P* < 0.001) and no worse than that in the image acquired with the reference radiation dose (Table 2 and Fig. 3D).

**Table 3:** Subject characteristics *in-vivo* study.

Variables	
Age(years)	66.3 ± 13.8
Female/male (*n*)	2/13
Body mass index	22.4 ± 3.97
Heart rate (bpm)	56.6 ± 7.00
R–R interval time (ms)	1,066 ± 147.7
CTDI (mGy)	50.2 ± 8.49
Contrast material volume (mL)	60.4 ± 13.0
Injection flow rate (mL/s)	3.60 ± 0.61

**Notes.**

CTDI computed tomography dose index

## Discussions

Our present study using static phantoms showed that *legato* could reduce noise throughout the investigated frequency band without degrading the CT numbers or spatial resolution. Further, the noise level of images obtained by scanning with a quarter radiation dose and post processing with *legato* was not inferior that of the reference images obtained with a full radiation dose.

CCT images provide a good test of the performance of *legato* in that several cardiac phases with short interval times are usually obtained as a padding phase (Tatsugami et al., 2015). In addition, the noise level would be higher than that in full reconstruction because there are fewer photons in the data acquisition of the half reconstruction that accounts for temporal resolution. Employing temporal filtering, this noise resulting from the half reconstruction could be compensated. Moreover, artifacts in half reconstruction are well known to mainly depend on the acquisition degree. Using a different phase means the use of a different acquisition degree. Thus, theoretically, these artifacts could also be minimized employed temporal noise reduction.

Interval times are important parameters when using *legato* in CCT. Small movements might be better for the precise estimation of movement. Thus, datasets with relative short interval times are required. However, it is not easy to distinguish noise in datasets for which the intervals are too short because the images and the noise they contain are almost the same. Our preliminary study showed that noise reduction for interval times from 10 to 30 ms was relatively low. In contrast, the noise reduction reached a plateau at interval times exceeding 50 ms. Larger interval times can thus improve noise reduction but possibly only to a certain degree. If the interval time is set as 70 ms, the three phases are separated by 90°for our scanner (having a rotation time of 280 ms). In dual-source CT, two detectors are aligned at almost 90°, and datasets having phases shifted by 90°contain data of completely different images with completely different acquisition. This would affect the limit of noise reduction of *legato* for CT images. Moreover, in coronary artery analysis, interval times of 50 ms rather than longer interval times are considered the most appropriate in a clinical setting. Because larger interval times mean departing from the mid-diastolic phase, these separated datasets could be adversely affected by a motion artifact of the coronary arteries. However, to investigate the appropriate interval times for *legato* in a clinical setting, further study using moving cardiac phantoms is needed. Moreover, the appropriate interval time would depend on the rotation speed and whether dual- or single-source CT was employed.

Image filtering can reduce the resolution and produce artifacts by oversmoothing. There is thus a trade-off between such artifacts and the reduced rate of noise. However, our *legato* can uphold the CT attenuation and the resolution with powerful noise reduction for static phantoms. Temporal filtering methods are known to avoid the oversmoothing of voxels because of their spatial independence (Brailean et al., 1995). Thus, *legato* improves the image quality by reducing noise while preserving structural detail, which is important for CCT. The importance of the spatial resolution has been well discussed in the detection of coronary stenosis (Leber et al., 2005). Recently, the CT attenuation of plaque has also been an important research topic. Plaque that is vulnerable to rupturing is known to have a low CT attenuation, and a small ulcer having a napkin-ring sign would be an important finding (Hoffmann et al., 2006; Motoyama et al., 2009). If the CT value changes, the definition of the plaque could be vague. Additionally, noise would easily affect these relatively low values. Noise-less images that maintain the CT attenuation and resolution would thus meet the clinical demands of CCT.

Our results also showed the possibility of combining *legato* with IR. Recently, powerful noise reduction using IR methods has been reported (Renker et al., 2011; Utsunomiya et al., 2012; Yin et al., 2013; Meyer et al., 2014). The IR methods are mainly used to reduce the radiation dose in clinical settings. Whereas the IR methods are used in the image reconstruction stage, *legato* is performed in the post-processing stage. Thus, *legato* could be applied to the IR images and further reduce noise independently of the IR methods.

Another possibility of *legato* is taking the same noise images with a lower radiation dose. Even at a quarter dose, the CNR obtained using *legato* was not less than that of the reference image. In addition, our subjective image quality assessment supports this finding. The results are thus reasonable. Without *legato*, the noise level would be twice that of the reference image for a quarter radiation dose. However, with *legato*, the noise level can be reduced by half, as previously discussed. Our result theoretically suggests that a three-quarter reduction of the radiation dose is possible when we focus on the image noise level, but a larger investigation of clinical cases is needed to assess the diagnostic performance.

There were limitations to the present study. First, in the *ex vivo* study, a static phantom was used to investigate the potential of *legato*. However, the effect of motion and the analysis of temporal resolution need to be further investigated for the use of CCT in clinical cases. Second, the application of *legato* to other scanners, such as a single-source scanner, and with other reconstruction kernels and other IR methods needs to be investigated.

In summary, our proposed 4DNR method can be applied to data series having short interval times (*legato*) to reduce noise without changing the CT attenuation or spatial resolution. Thus, applying this post-processing method to the “padding” phases of CCT images would have a potential of theoretically achieving a 75% reduction at the maximum in the radiation dose while maintaining the noise level as that of the baseline radiation dose. However, further studies are needed for the clinical usage.

## Abbreviations

CT: Computed tomography
CCT: Cardiac CT
CNR: Contrast-to-noise ratio
4DNR: Four- dimensional noise reduction
FBP: Filtered back projection
IR: Iterative reconstruction
MTF: Modulation transfer function
NPS: Noise power spectrum
*Legato*: The method of obtaining a noisele ss imag e by a daptive phase-shifted to pological coherence analysis

## Additional Information and Declarations

### Data Availability

The following information was supplied regarding data availability: Figshare: http://figshare.com/s/c6fda35678aa11e5b50906ec4b8d1f61.
